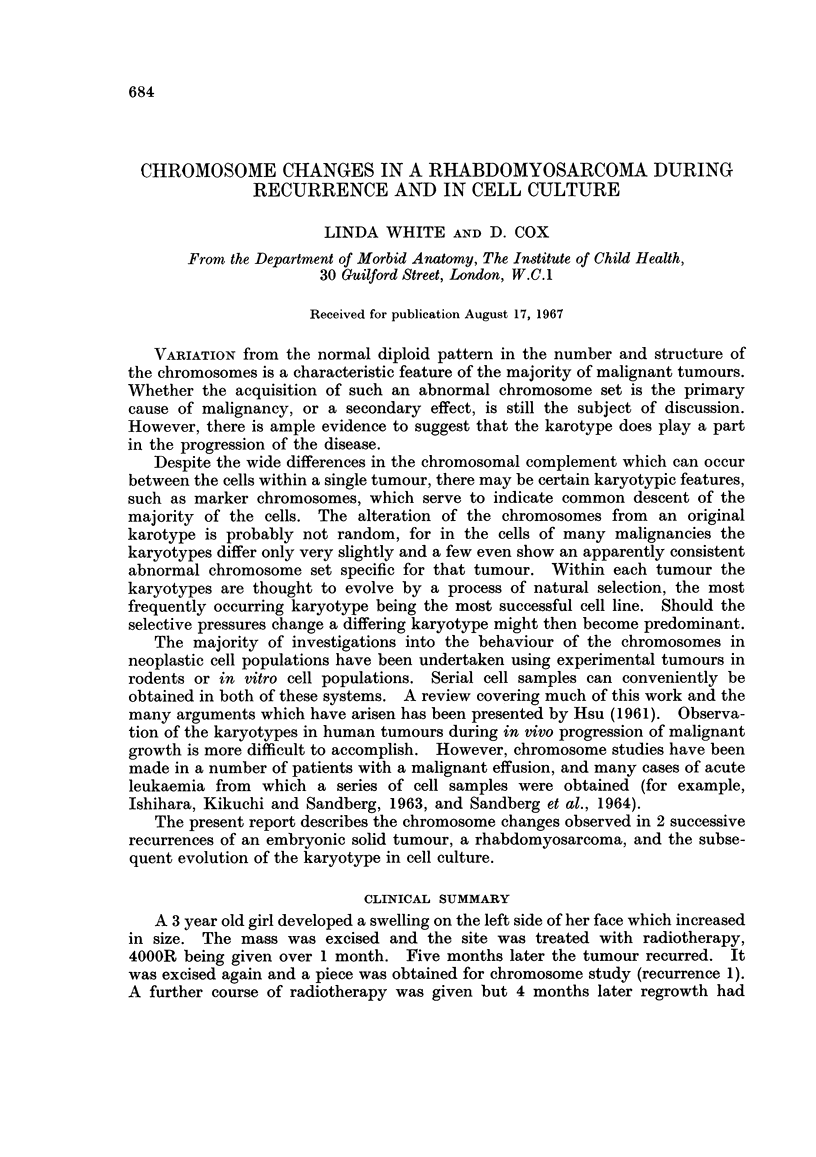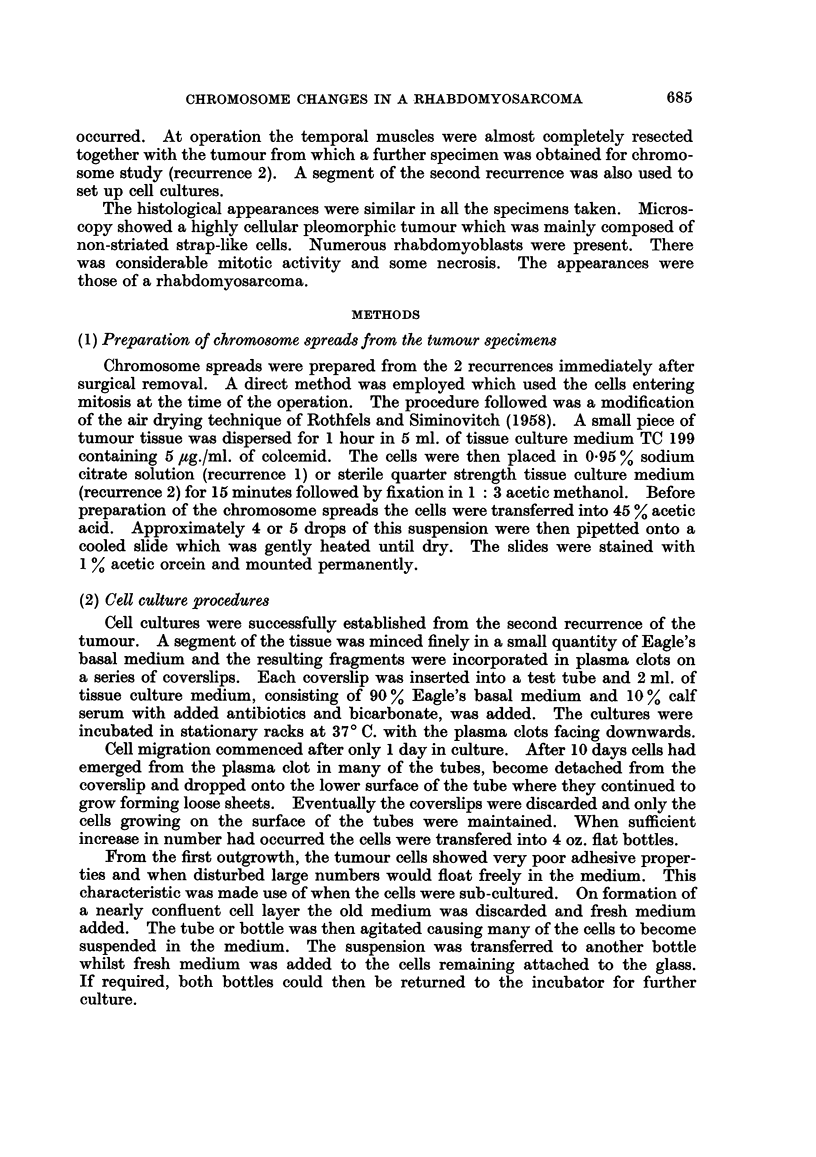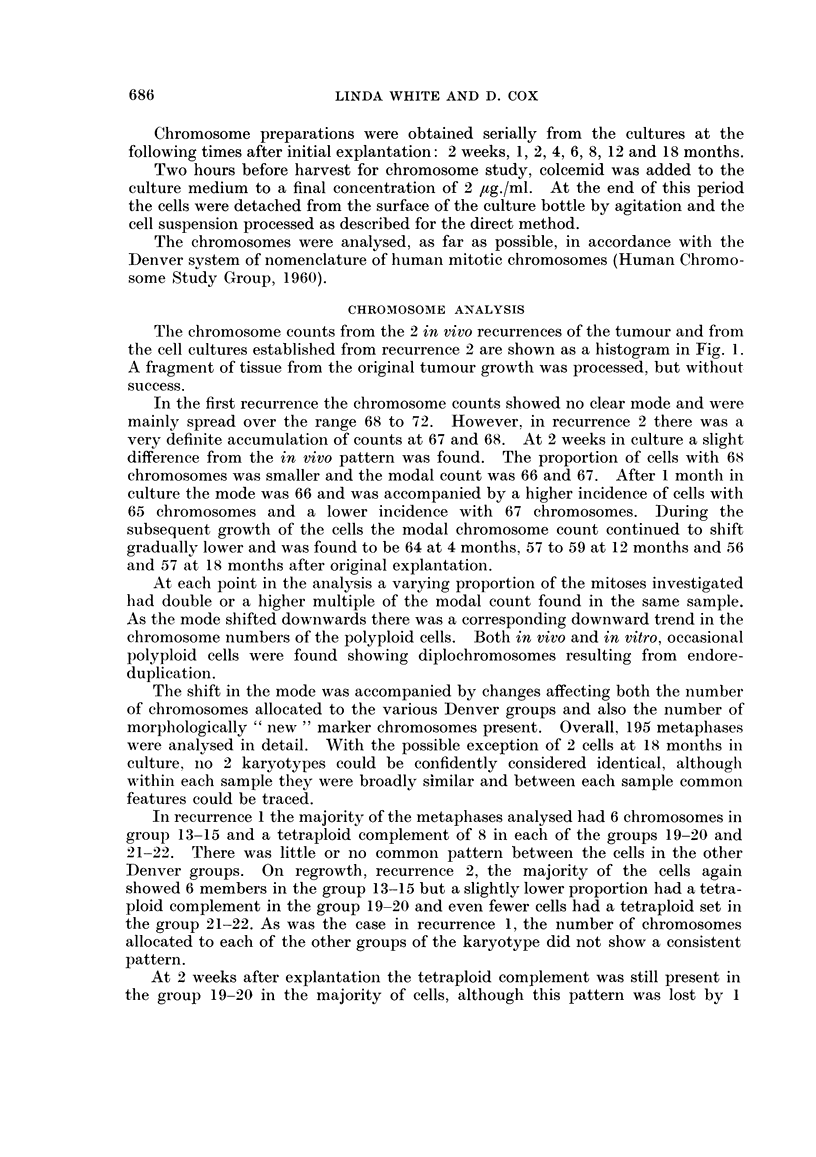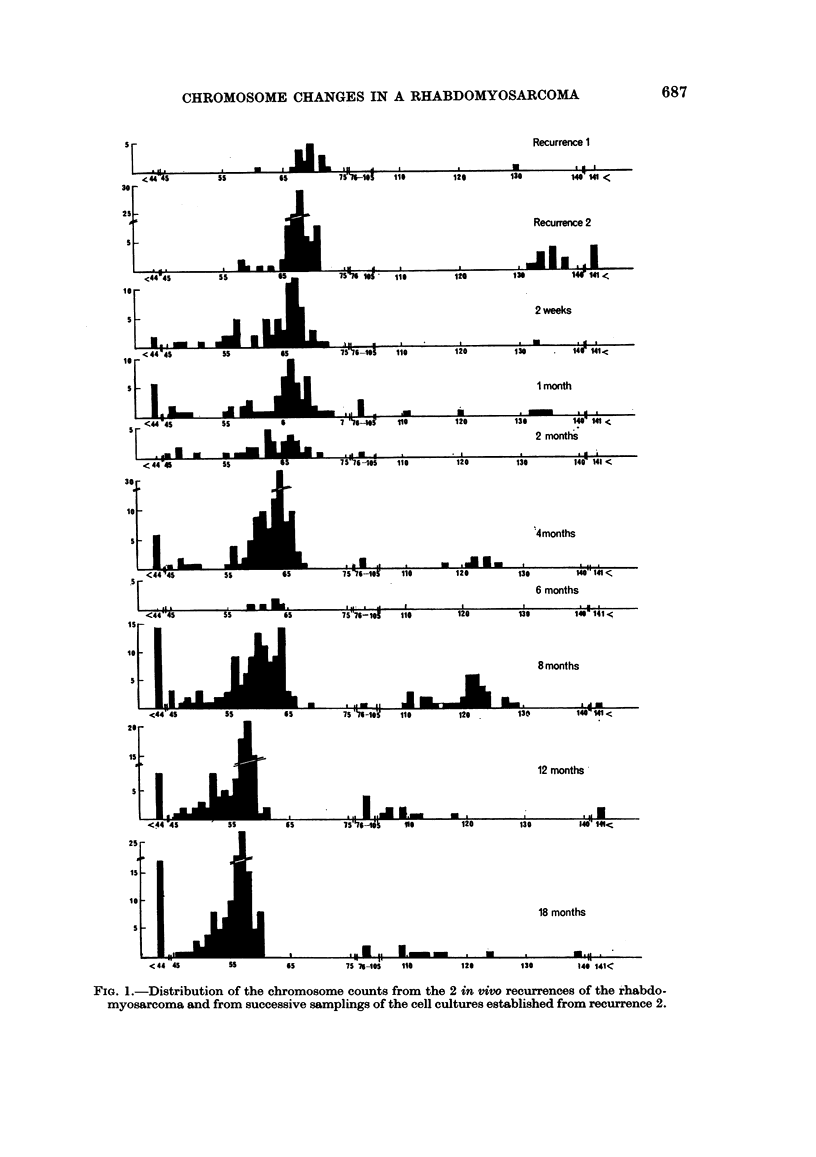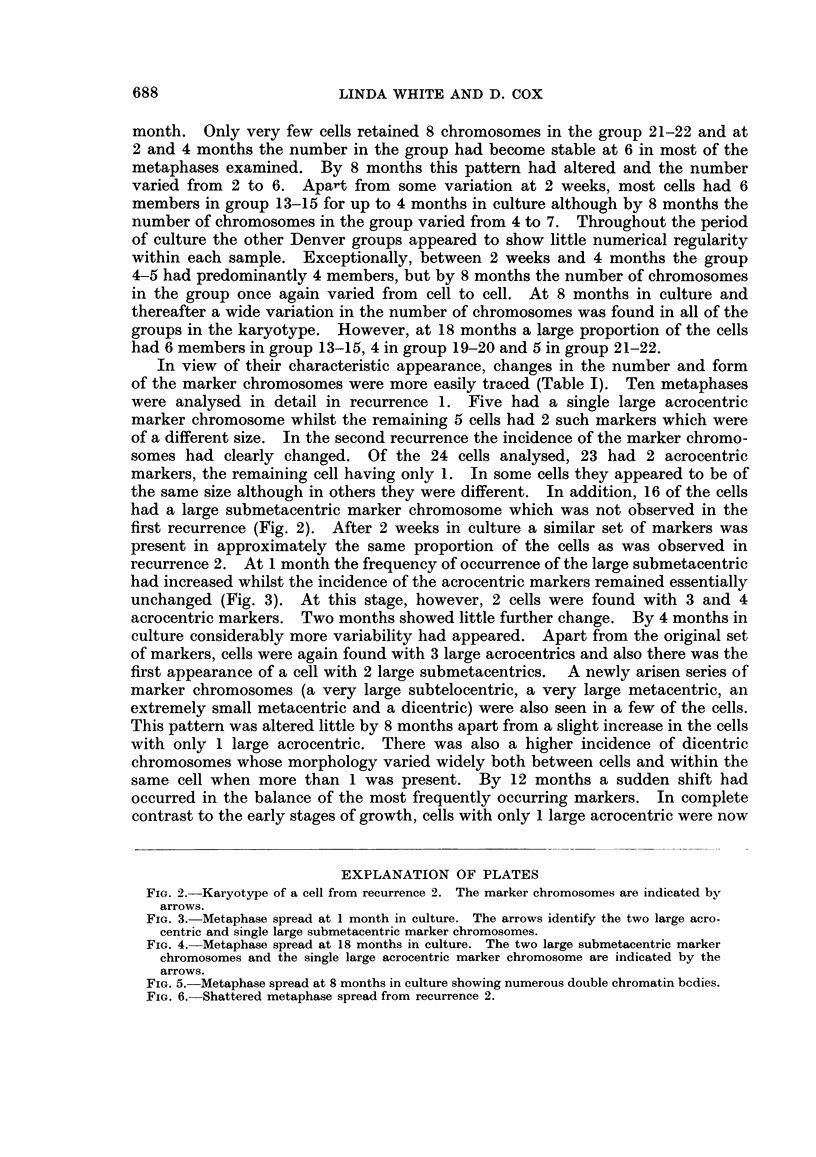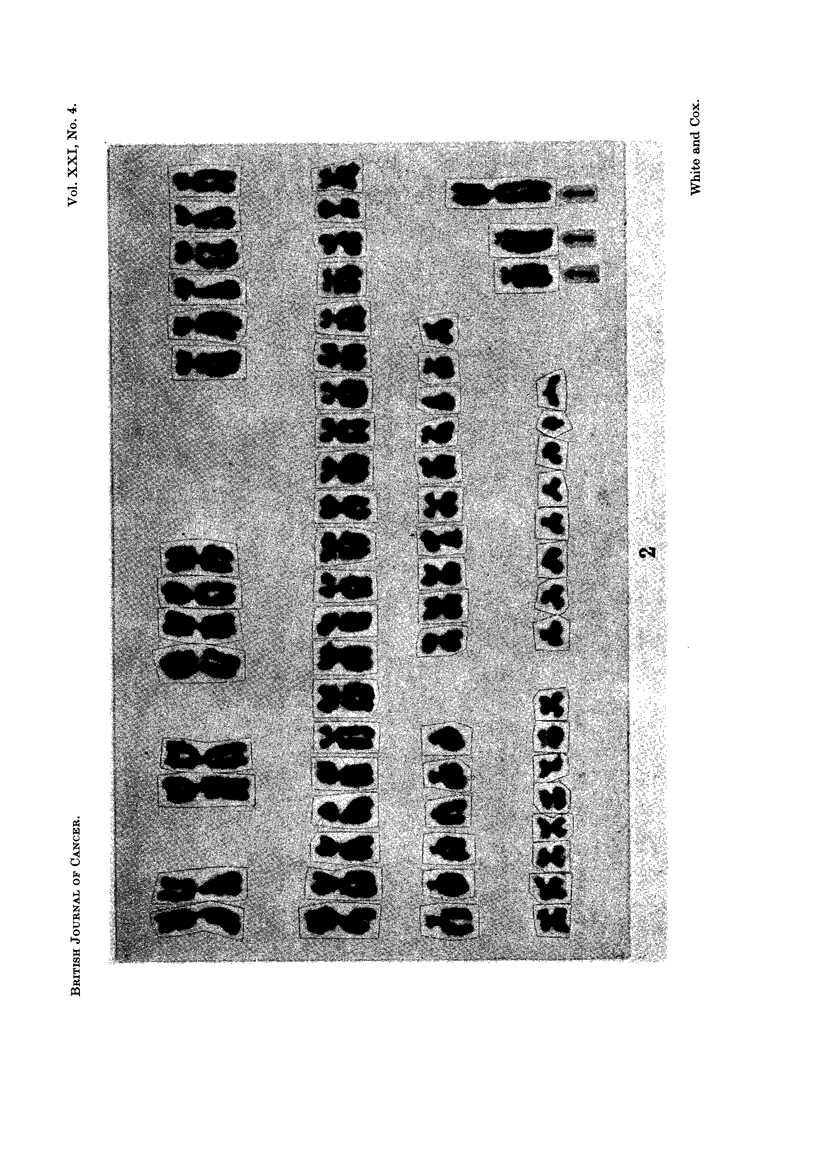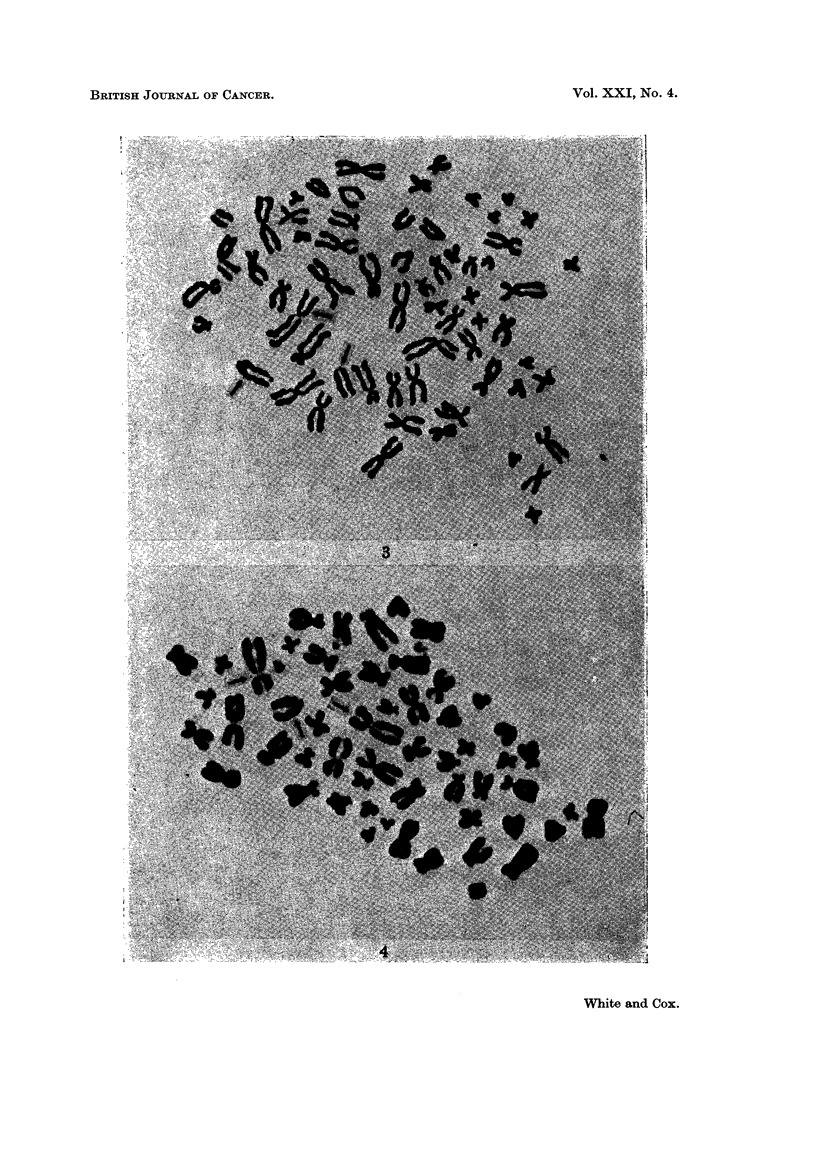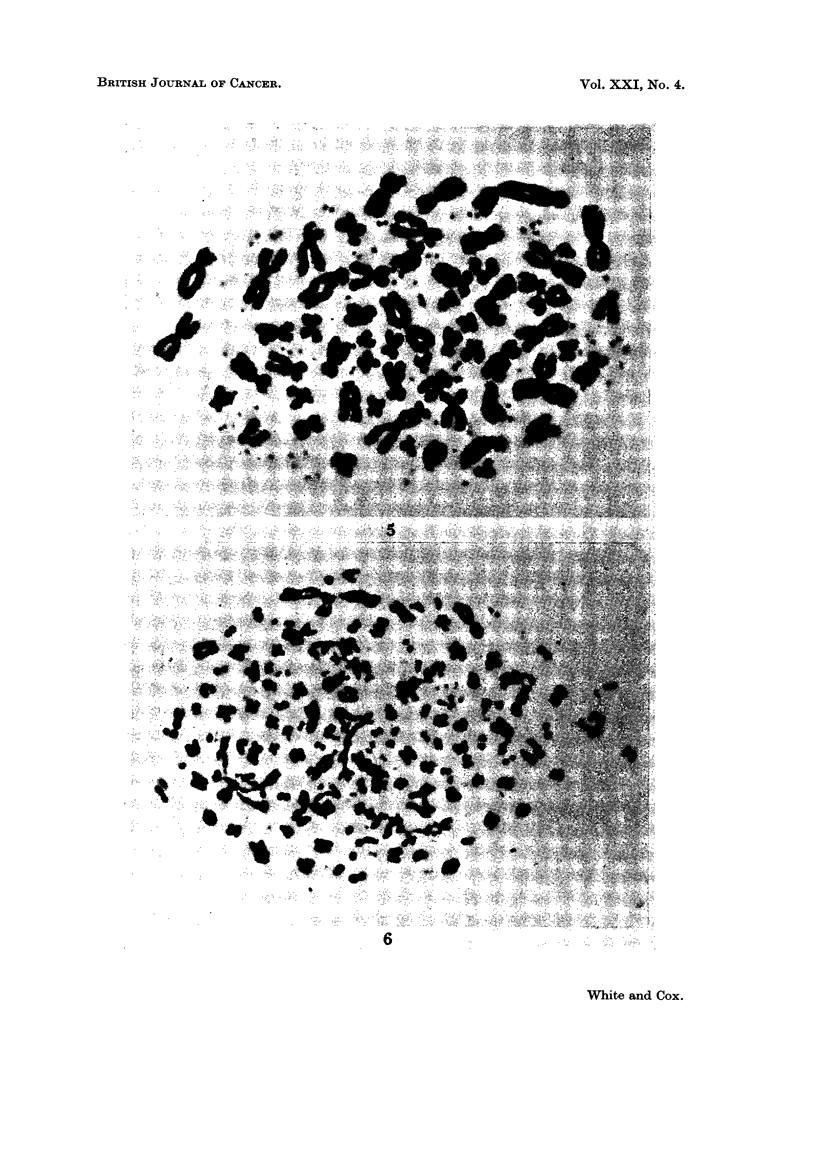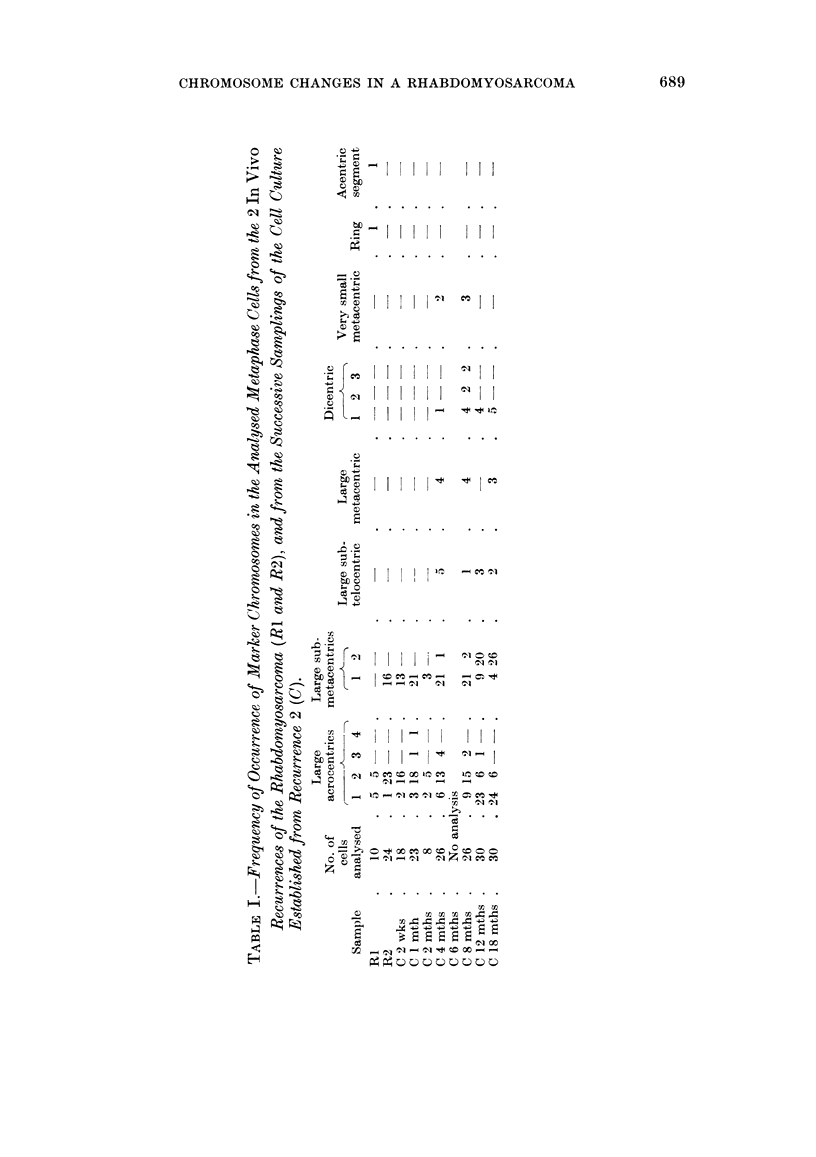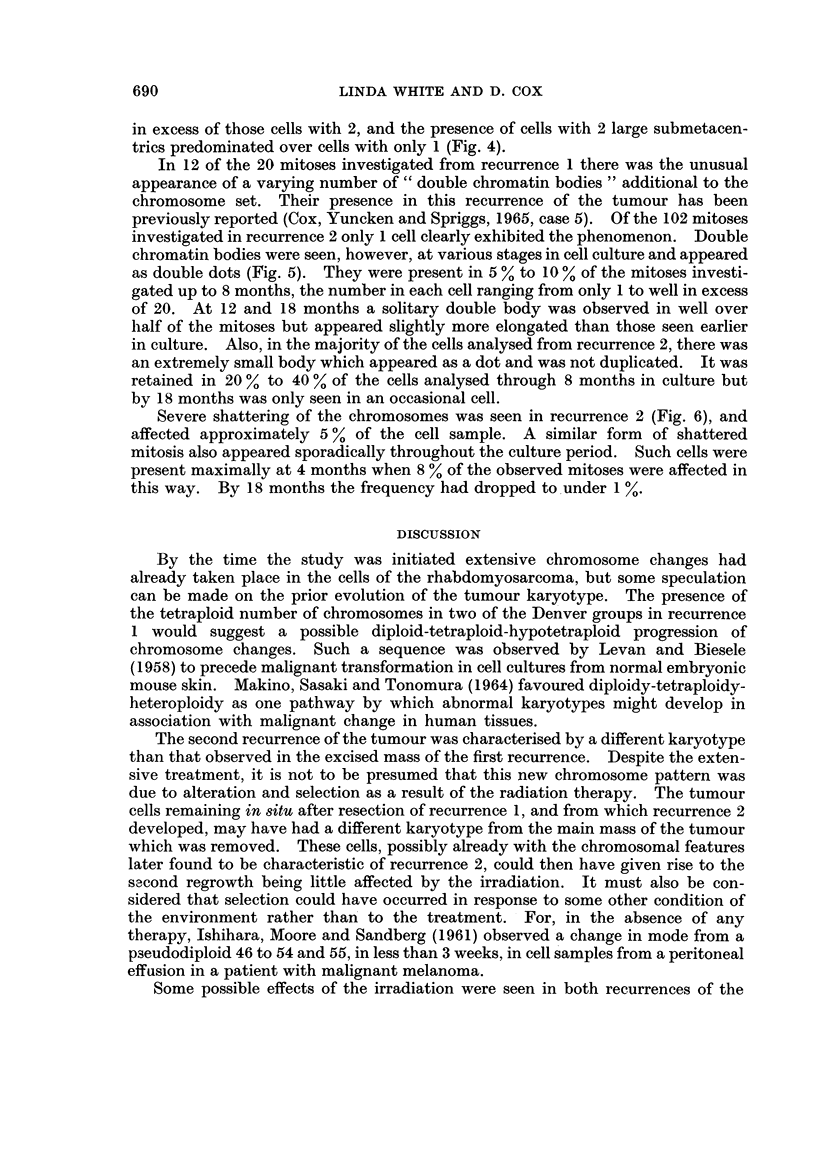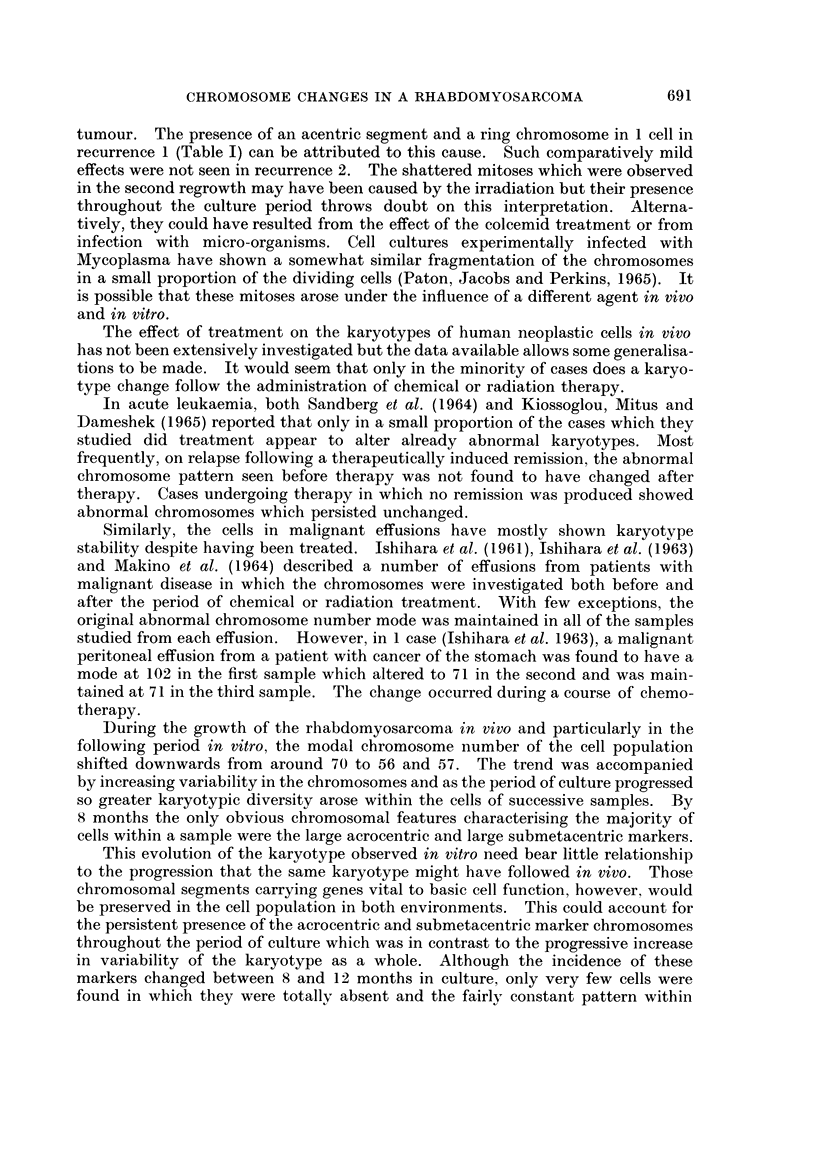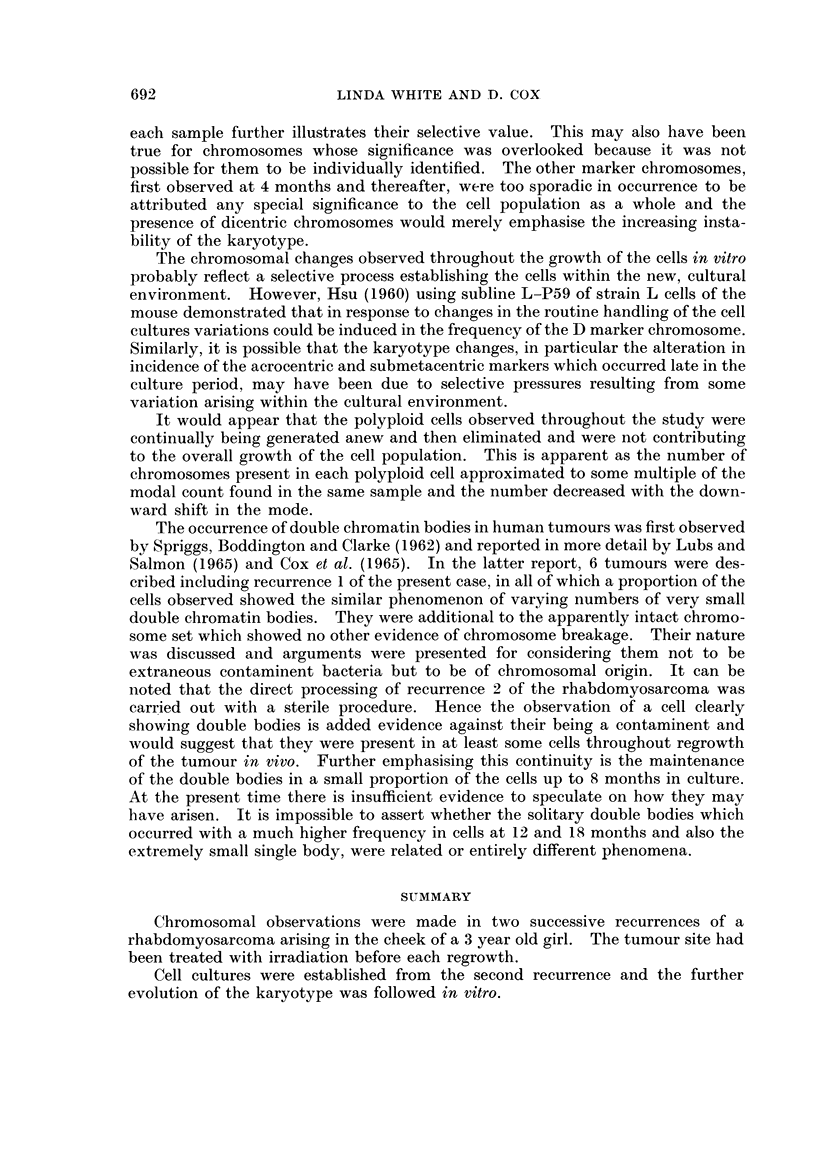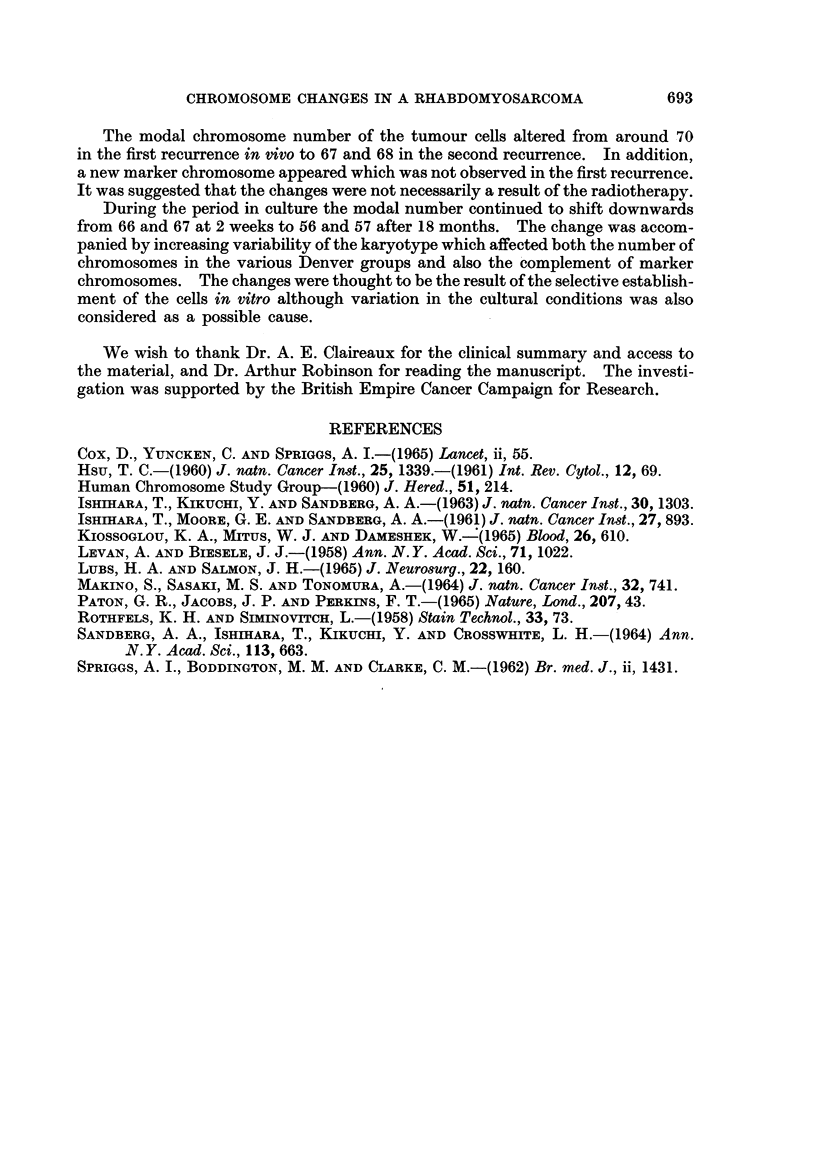# Chromosome changes in a rhabdomyosarcoma during recurrence and in cell culture.

**DOI:** 10.1038/bjc.1967.81

**Published:** 1967-12

**Authors:** L. White, D. Cox

## Abstract

**Images:**


### CHROMOSOME CHANGES IN A RHABDOMYOSARCOMA DURING RECURRENCE AND IN CELL CULTURE

LINDA WHITE AND D. COX

*From the Department of Morbid Anatomy, The Institute of Child Health, 30 Guilford Street, London, W.C.1*

Received for publication August 17, 1967

VARIATION from the normal diploid pattern in the number and structure of the chromosomes is a characteristic feature of the majority of malignant tumours. Whether the acquisition of such an abnormal chromosome set is the primary cause of malignancy, or a secondary effect, is still the subject of discussion. However, there is ample evidence to suggest that the karotype does play a part in the progression of the disease.

Despite the wide differences in the chromosomal complement which can occur between the cells within a single tumour, there may be certain karyotypic features, such as marker chromosomes, which serve to indicate common descent of the majority of the cells. The alteration of the chromosomes from an original karotype is probably not random, for in the cells of many malignancies the karyotypes differ only very slightly and a few even show an apparently consistent abnormal chromosome set specific for that tumour. Within each tumour the karyotypes are thought to evolve by a process of natural selection, the most frequently occurring karyotype being the most successful cell line. Should the selective pressures change a differing karyotype might then become predominant.

The majority of investigations into the behaviour of the chromosomes in neoplastic cell populations have been undertaken using experimental tumours in rodents or *in vitro* cell populations. Serial cell samples can conveniently be obtained in both of these systems. A review covering much of this work and the many arguments which have arisen has been presented by Hsu (1961). Observation of the karyotypes in human tumours during *in vivo* progression of malignant growth is more difficult to accomplish. However, chromosome studies have been made in a number of patients with a malignant effusion, and many cases of acute leukaemia from which a series of cell samples were obtained (for example, Ishihara, Kikuchi and Sandberg, 1963, and Sandberg *et al.*, 1964).

The present report describes the chromosome changes observed in 2 successive recurrences of an embryonic solid tumour, a rhabdomyosarcoma, and the subsequent evolution of the karyotype in cell culture.

### CLINICAL SUMMARY

A 3 year old girl developed a swelling on the left side of her face which increased in size. The mass was excised and the site was treated with radiotherapy, 4000R being given over 1 month. Five months later the tumour recurred. It was excised again and a piece was obtained for chromosome study (recurrence 1). A further course of radiotherapy was given but 4 months later regrowth had occurred. At operation the temporal muscles were almost completely resected together with the tumour from which a further specimen was obtained for chromosome study (recurrence 2). A segment of the second recurrence was also used to set up cell cultures.

The histological appearances were similar in all the specimens taken. Microscopy showed a highly cellular pleomorphic tumour which was mainly composed of non-striated strap-like cells. Numerous rhabdomyoblasts were present. There was considerable mitotic activity and some necrosis. The appearances were those of a rhabdomyosarcoma.

### METHODS

### (1) *Preparation of chromosome spreads from the tumour specimens*

Chromosome spreads were prepared from the 2 recurrences immediately after surgical removal. A direct method was employed which used the cells entering mitosis at the time of the operation. The procedure followed was a modification of the air drying technique of Rothfels and Siminovitch (1958). A small piece of tumour tissue was dispersed for 1 hour in 5 ml. of tissue culture medium TC 199 containing 5 $\mu$g./ml. of colcemid. The cells were then placed in 0·95 % sodium citrate solution (recurrence 1) or sterile quarter strength tissue culture medium (recurrence 2) for 15 minutes followed by fixation in 1 : 3 acetic methanol. Before preparation of the chromosome spreads the cells were transferred into 45 % acetic acid. Approximately 4 or 5 drops of this suspension were then pipetted onto a cooled slide which was gently heated until dry. The slides were stained with 1 % acetic orcein and mounted permanently.

### (2) *Cell culture procedures*

Cell cultures were successfully established from the second recurrence of the tumour. A segment of the tissue was minced finely in a small quantity of Eagle's basal medium and the resulting fragments were incorporated in plasma clots on a series of coverslips. Each coverslip was inserted into a test tube and 2 ml. of tissue culture medium, consisting of 90 % Eagle's basal medium and 10 % calf serum with added antibiotics and bicarbonate, was added. The cultures were incubated in stationary racks at 37° C. with the plasma clots facing downwards.

Cell migration commenced after only 1 day in culture. After 10 days cells had emerged from the plasma clot in many of the tubes, become detached from the coverslip and dropped onto the lower surface of the tube where they continued to grow forming loose sheets. Eventually the coverslips were discarded and only the cells growing on the surface of the tubes were maintained. When sufficient increase in number had occurred the cells were transfered into 4 oz. flat bottles.

From the first outgrowth, the tumour cells showed very poor adhesive properties and when disturbed large numbers would float freely in the medium. This characteristic was made use of when the cells were sub-cultured. On formation of a nearly confluent cell layer the old medium was discarded and fresh medium added. The tube or bottle was then agitated causing many of the cells to become suspended in the medium. The suspension was transferred to another bottle whilst fresh medium was added to the cells remaining attached to the glass. If required, both bottles could then be returned to the incubator for further culture.

Chromosome preparations were obtained serially from the cultures at the following times after initial explantation: 2 weeks, 1, 2, 4, 6, 8, 12 and 18 months.

Two hours before harvest for chromosome study, colcemid was added to the culture medium to a final concentration of 2 $\mu$g./ml. At the end of this period the cells were detached from the surface of the culture bottle by agitation and the cell suspension processed as described for the direct method.

The chromosomes were analysed, as far as possible, in accordance with the Denver system of nomenclature of human mitotic chromosomes (Human Chromosome Study Group, 1960).

### CHROMOSOME ANALYSIS

The chromosome counts from the 2 *in vivo* recurrences of the tumour and from the cell cultures established from recurrence 2 are shown as a histogram in Fig. 1. A fragment of tissue from the original tumour growth was processed, but without success.

In the first recurrence the chromosome counts showed no clear mode and were mainly spread over the range 68 to 72. However, in recurrence 2 there was a very definite accumulation of counts at 67 and 68. At 2 weeks in culture a slight difference from the *in vivo* pattern was found. The proportion of cells with 68 chromosomes was smaller and the modal count was 66 and 67. After 1 month in culture the mode was 66 and was accompanied by a higher incidence of cells with 65 chromosomes and a lower incidence with 67 chromosomes. During the subsequent growth of the cells the modal chromosome count continued to shift gradually lower and was found to be 64 at 4 months, 57 to 59 at 12 months and 56 and 57 at 18 months after original explantation.

At each point in the analysis a varying proportion of the mitoses investigated had double or a higher multiple of the modal count found in the same sample. As the mode shifted downwards there was a corresponding downward trend in the chromosome numbers of the polyploid cells. Both *in vivo* and *in vitro*, occasional polyploid cells were found showing diplochromosomes resulting from endoreduplication.

The shift in the mode was accompanied by changes affecting both the number of chromosomes allocated to the various Denver groups and also the number of morphologically " new " marker chromosomes present. Overall, 195 metaphases were analysed in detail. With the possible exception of 2 cells at 18 months in culture, no 2 karyotypes could be confidently considered identical, although within each sample they were broadly similar and between each sample common features could be traced.

In recurrence 1 the majority of the metaphases analysed had 6 chromosomes in group 13–15 and a tetraploid complement of 8 in each of the groups 19–20 and 21–22. There was little or no common pattern between the cells in the other Denver groups. On regrowth, recurrence 2, the majority of the cells again showed 6 members in the group 13–15 but a slightly lower proportion had a tetraploid complement in the group 19–20 and even fewer cells had a tetraploid set in the group 21–22. As was the case in recurrence 1, the number of chromosomes allocated to each of the other groups of the karyotype did not show a consistent pattern.

At 2 weeks after explantation the tetraploid complement was still present in the group 19–20 in the majority of cells, although this pattern was lost by 1

FIG. 1.—Distribution of the chromosome counts from the 2 *in vivo* recurrences of the rhabdomyosarcoma and from successive samplings of the cell cultures established from recurrence 2.

month. Only very few cells retained 8 chromosomes in the group 21–22 and at 2 and 4 months the number in the group had become stable at 6 in most of the metaphases examined. By 8 months this pattern had altered and the number varied from 2 to 6. Apart from some variation at 2 weeks, most cells had 6 members in group 13–15 for up to 4 months in culture although by 8 months the number of chromosomes in the group varied from 4 to 7. Throughout the period of culture the other Denver groups appeared to show little numerical regularity within each sample. Exceptionally, between 2 weeks and 4 months the group 4–5 had predominantly 4 members, but by 8 months the number of chromosomes in the group once again varied from cell to cell. At 8 months in culture and thereafter a wide variation in the number of chromosomes was found in all of the groups in the karyotype. However, at 18 months a large proportion of the cells had 6 members in group 13–15, 4 in group 19–20 and 5 in group 21–22.

In view of their characteristic appearance, changes in the number and form of the marker chromosomes were more easily traced (Table I). Ten metaphases were analysed in detail in recurrence 1. Five had a single large acrocentric marker chromosome whilst the remaining 5 cells had 2 such markers which were of a different size. In the second recurrence the incidence of the marker chromosomes had clearly changed. Of the 24 cells analysed, 23 had 2 acrocentric markers, the remaining cell having only 1. In some cells they appeared to be of the same size although in others they were different. In addition, 16 of the cells had a large submetacentric marker chromosome which was not observed in the first recurrence (Fig. 2). After 2 weeks in culture a similar set of markers was present in approximately the same proportion of the cells as was observed in recurrence 2. At 1 month the frequency of occurrence of the large submetacentric had increased whilst the incidence of the acrocentric markers remained essentially unchanged (Fig. 3). At this stage, however, 2 cells were found with 3 and 4 acrocentric markers. Two months showed little further change. By 4 months in culture considerably more variability had appeared. Apart from the original set of markers, cells were again found with 3 large acrocentrics and also there was the first appearance of a cell with 2 large submetacentrics. A newly arisen series of marker chromosomes (a very large subtelocentric, a very large metacentric, an extremely small metacentric and a dicentric) were also seen in a few of the cells. This pattern was altered little by 8 months apart from a slight increase in the cells with only 1 large acrocentric. There was also a higher incidence of dicentric chromosomes whose morphology varied widely both between cells and within the same cell when more than 1 was present. By 12 months a sudden shift had occurred in the balance of the most frequently occurring markers. In complete contrast to the early stages of growth, cells with only 1 large acrocentric were now

---

EXPLANATION OF PLATES

FIG. 2.—Karyotype of a cell from recurrence 2. The marker chromosomes are indicated by arrows.

FIG. 3.—Metaphase spread at 1 month in culture. The arrows identify the two large acrocentric and single large submetacentric marker chromosomes.

FIG. 4.—Metaphase spread at 18 months in culture. The two large submetacentric marker chromosomes and the single large acrocentric marker chromosome are indicated by the arrows.

FIG. 5.—Metaphase spread at 8 months in culture showing numerous double chromatin bodies.

FIG. 6.—Shattered metaphase spread from recurrence 2.

2

White and Cox.

3

4

5

6

White and Cox.

TABLE I.—*Frequency of Occurrence of Marker Chromosomes in the Analysed Metaphase Cells from the* 2 In Vivo *Recurrences of the Rhabdomyosarcoma* (*R*1 *and R*2), *and from the Successive Samplings of the Cell Culture Established from Recurrence* 2 (*C*).

| Sample | No. of cells analysed | Large acrocentrics 1 | 2 | 3 | 4 | Large sub-metacentrics 1 | 2 | Large sub-telocentric | Large metacentric | Dicentric 1 | 2 | 3 | Very small metacentric | Ring | Acentric segment |
|---|---|---|---|---|---|---|---|---|---|---|---|---|---|---|---|
| R1 | 10 | 5 | 5 | — | — | — | — | — | — | — | — | — | — | 1 | 1 |
| R2 | 24 | 1 | 23 | — | — | 16 | — | — | — | — | — | — | — | — | — |
| C 2 wks | 18 | 2 | 16 | — | — | 13 | — | — | — | — | — | — | — | — | — |
| C 1 mth | 23 | 3 | 18 | 1 | 1 | 21 | — | — | — | — | — | — | — | — | — |
| C 2 mths | 8 | 2 | 5 | — | — | 3 | — | — | — | — | — | — | — | — | — |
| C 4 mths | 26 | 6 | 13 | 4 | — | 21 | 1 | 5 | 4 | 1 | — | — | 2 | — | — |
| C 6 mths | No analysis | | | | | | | | | | | | | | |
| C 8 mths | 26 | 9 | 15 | 2 | — | 21 | 2 | 1 | 4 | 4 | 2 | 2 | 3 | — | — |
| C 12 mths | 30 | 23 | 6 | 1 | — | 9 | 20 | 3 | — | 4 | — | — | — | — | — |
| C 18 mths | 30 | 24 | 6 | — | — | 4 | 26 | 2 | 3 | 5 | — | — | — | — | — |

in excess of those cells with 2, and the presence of cells with 2 large submetacentrics predominated over cells with only 1 (Fig. 4).

In 12 of the 20 mitoses investigated from recurrence 1 there was the unusual appearance of a varying number of " double chromatin bodies " additional to the chromosome set. Their presence in this recurrence of the tumour has been previously reported (Cox, Yuncken and Spriggs, 1965, case 5). Of the 102 mitoses investigated in recurrence 2 only 1 cell clearly exhibited the phenomenon. Double chromatin bodies were seen, however, at various stages in cell culture and appeared as double dots (Fig. 5). They were present in 5 % to 10 % of the mitoses investigated up to 8 months, the number in each cell ranging from only 1 to well in excess of 20. At 12 and 18 months a solitary double body was observed in well over half of the mitoses but appeared slightly more elongated than those seen earlier in culture. Also, in the majority of the cells analysed from recurrence 2, there was an extremely small body which appeared as a dot and was not duplicated. It was retained in 20 % to 40 % of the cells analysed through 8 months in culture but by 18 months was only seen in an occasional cell.

Severe shattering of the chromosomes was seen in recurrence 2 (Fig. 6), and affected approximately 5 % of the cell sample. A similar form of shattered mitosis also appeared sporadically throughout the culture period. Such cells were present maximally at 4 months when 8 % of the observed mitoses were affected in this way. By 18 months the frequency had dropped to under 1 %.

### DISCUSSION

By the time the study was initiated extensive chromosome changes had already taken place in the cells of the rhabdomyosarcoma, but some speculation can be made on the prior evolution of the tumour karyotype. The presence of the tetraploid number of chromosomes in two of the Denver groups in recurrence 1 would suggest a possible diploid-tetraploid-hypotetraploid progression of chromosome changes. Such a sequence was observed by Levan and Biesele (1958) to precede malignant transformation in cell cultures from normal embryonic mouse skin. Makino, Sasaki and Tonomura (1964) favoured diploidy-tetraploidy-heteroploidy as one pathway by which abnormal karyotypes might develop in association with malignant change in human tissues.

The second recurrence of the tumour was characterised by a different karyotype than that observed in the excised mass of the first recurrence. Despite the extensive treatment, it is not to be presumed that this new chromosome pattern was due to alteration and selection as a result of the radiation therapy. The tumour cells remaining *in situ* after resection of recurrence 1, and from which recurrence 2 developed, may have had a different karyotype from the main mass of the tumour which was removed. These cells, possibly already with the chromosomal features later found to be characteristic of recurrence 2, could then have given rise to the second regrowth being little affected by the irradiation. It must also be considered that selection could have occurred in response to some other condition of the environment rather than to the treatment. For, in the absence of any therapy, Ishihara, Moore and Sandberg (1961) observed a change in mode from a pseudodiploid 46 to 54 and 55, in less than 3 weeks, in cell samples from a peritoneal effusion in a patient with malignant melanoma.

Some possible effects of the irradiation were seen in both recurrences of the tumour. The presence of an acentric segment and a ring chromosome in 1 cell in recurrence 1 (Table I) can be attributed to this cause. Such comparatively mild effects were not seen in recurrence 2. The shattered mitoses which were observed in the second regrowth may have been caused by the irradiation but their presence throughout the culture period throws doubt on this interpretation. Alternatively, they could have resulted from the effect of the colcemid treatment or from infection with micro-organisms. Cell cultures experimentally infected with Mycoplasma have shown a somewhat similar fragmentation of the chromosomes in a small proportion of the dividing cells (Paton, Jacobs and Perkins, 1965). It is possible that these mitoses arose under the influence of a different agent *in vivo* and *in vitro*.

The effect of treatment on the karyotypes of human neoplastic cells *in vivo* has not been extensively investigated but the data available allows some generalisations to be made. It would seem that only in the minority of cases does a karyotype change follow the administration of chemical or radiation therapy.

In acute leukaemia, both Sandberg *et al.* (1964) and Kiossoglou, Mitus and Dameshek (1965) reported that only in a small proportion of the cases which they studied did treatment appear to alter already abnormal karyotypes. Most frequently, on relapse following a therapeutically induced remission, the abnormal chromosome pattern seen before therapy was not found to have changed after therapy. Cases undergoing therapy in which no remission was produced showed abnormal chromosomes which persisted unchanged.

Similarly, the cells in malignant effusions have mostly shown karyotype stability despite having been treated. Ishihara *et al.* (1961), Ishihara *et al.* (1963) and Makino *et al.* (1964) described a number of effusions from patients with malignant disease in which the chromosomes were investigated both before and after the period of chemical or radiation treatment. With few exceptions, the original abnormal chromosome number mode was maintained in all of the samples studied from each effusion. However, in 1 case (Ishihara *et al.* 1963), a malignant peritoneal effusion from a patient with cancer of the stomach was found to have a mode at 102 in the first sample which altered to 71 in the second and was maintained at 71 in the third sample. The change occurred during a course of chemotherapy.

During the growth of the rhabdomyosarcoma *in vivo* and particularly in the following period *in vitro*, the modal chromosome number of the cell population shifted downwards from around 70 to 56 and 57. The trend was accompanied by increasing variability in the chromosomes and as the period of culture progressed so greater karyotypic diversity arose within the cells of successive samples. By 8 months the only obvious chromosomal features characterising the majority of cells within a sample were the large acrocentric and large submetacentric markers.

This evolution of the karyotype observed *in vitro* need bear little relationship to the progression that the same karyotype might have followed *in vivo*. Those chromosomal segments carrying genes vital to basic cell function, however, would be preserved in the cell population in both environments. This could account for the persistent presence of the acrocentric and submetacentric marker chromosomes throughout the period of culture which was in contrast to the progressive increase in variability of the karyotype as a whole. Although the incidence of these markers changed between 8 and 12 months in culture, only very few cells were found in which they were totally absent and the fairly constant pattern within each sample further illustrates their selective value. This may also have been true for chromosomes whose significance was overlooked because it was not possible for them to be individually identified. The other marker chromosomes, first observed at 4 months and thereafter, were too sporadic in occurrence to be attributed any special significance to the cell population as a whole and the presence of dicentric chromosomes would merely emphasise the increasing instability of the karyotype.

The chromosomal changes observed throughout the growth of the cells *in vitro* probably reflect a selective process establishing the cells within the new, cultural environment. However, Hsu (1960) using subline L–P59 of strain L cells of the mouse demonstrated that in response to changes in the routine handling of the cell cultures variations could be induced in the frequency of the D marker chromosome. Similarly, it is possible that the karyotype changes, in particular the alteration in incidence of the acrocentric and submetacentric markers which occurred late in the culture period, may have been due to selective pressures resulting from some variation arising within the cultural environment.

It would appear that the polyploid cells observed throughout the study were continually being generated anew and then eliminated and were not contributing to the overall growth of the cell population. This is apparent as the number of chromosomes present in each polyploid cell approximated to some multiple of the modal count found in the same sample and the number decreased with the downward shift in the mode.

The occurrence of double chromatin bodies in human tumours was first observed by Spriggs, Boddington and Clarke (1962) and reported in more detail by Lubs and Salmon (1965) and Cox *et al.* (1965). In the latter report, 6 tumours were described including recurrence 1 of the present case, in all of which a proportion of the cells observed showed the similar phenomenon of varying numbers of very small double chromatin bodies. They were additional to the apparently intact chromosome set which showed no other evidence of chromosome breakage. Their nature was discussed and arguments were presented for considering them not to be extraneous contaminent bacteria but to be of chromosomal origin. It can be noted that the direct processing of recurrence 2 of the rhabdomyosarcoma was carried out with a sterile procedure. Hence the observation of a cell clearly showing double bodies is added evidence against their being a contaminent and would suggest that they were present in at least some cells throughout regrowth of the tumour *in vivo*. Further emphasising this continuity is the maintenance of the double bodies in a small proportion of the cells up to 8 months in culture. At the present time there is insufficient evidence to speculate on how they may have arisen. It is impossible to assert whether the solitary double bodies which occurred with a much higher frequency in cells at 12 and 18 months and also the extremely small single body, were related or entirely different phenomena.

### SUMMARY

Chromosomal observations were made in two successive recurrences of a rhabdomyosarcoma arising in the cheek of a 3 year old girl. The tumour site had been treated with irradiation before each regrowth.

Cell cultures were established from the second recurrence and the further evolution of the karyotype was followed *in vitro*.

The modal chromosome number of the tumour cells altered from around 70 in the first recurrence *in vivo* to 67 and 68 in the second recurrence. In addition, a new marker chromosome appeared which was not observed in the first recurrence. It was suggested that the changes were not necessarily a result of the radiotherapy.

During the period in culture the modal number continued to shift downwards from 66 and 67 at 2 weeks to 56 and 57 after 18 months. The change was accompanied by increasing variability of the karyotype which affected both the number of chromosomes in the various Denver groups and also the complement of marker chromosomes. The changes were thought to be the result of the selective establishment of the cells *in vitro* although variation in the cultural conditions was also considered as a possible cause.

We wish to thank Dr. A. E. Claireaux for the clinical summary and access to the material, and Dr. Arthur Robinson for reading the manuscript. The investigation was supported by the British Empire Cancer Campaign for Research.

### REFERENCES

Cox, D., Yuncken, C. and Spriggs, A. I.—(1965) *Lancet*, ii, 55.
Hsu, T. C.—(1960) *J. natn. Cancer Inst.*, **25,** 1339.—(1961) *Int. Rev. Cytol.*, **12,** 69.
Human Chromosome Study Group—(1960) *J. Hered.*, **51,** 214.
Ishihara, T., Kikuchi, Y. and Sandberg, A. A.—(1963) *J. natn. Cancer Inst.*, **30,** 1303.
Ishihara, T., Moore, G. E. and Sandberg, A. A.—(1961) *J. natn. Cancer Inst.*, **27,** 893.
Kiossoglou, K. A., Mitus, W. J. and Dameshek, W.—(1965) *Blood*, **26,** 610.
Levan, A. and Biesele, J. J.—(1958) *Ann. N.Y. Acad. Sci.*, **71,** 1022.
Lubs, H. A. and Salmon, J. H.—(1965) *J. Neurosurg.*, **22,** 160.
Makino, S., Sasaki, M. S. and Tonomura, A.—(1964) *J. natn. Cancer Inst.*, **32,** 741.
Paton, G. R., Jacobs, J. P. and Perkins, F. T.—(1965) *Nature, Lond.*, **207,** 43.
Rothfels, K. H. and Siminovitch, L.—(1958) *Stain Technol.*, **33,** 73.
Sandberg, A. A., Ishihara, T., Kikuchi, Y. and Crosswhite, L. H.—(1964) *Ann. N.Y. Acad. Sci.*, **113,** 663.
Spriggs, A. I., Boddington, M. M. and Clarke, C. M.—(1962) *Br. med. J.*, ii, 1431.